# Harnessing artificial intelligence for prostate cancer management

**DOI:** 10.1016/j.xcrm.2024.101506

**Published:** 2024-04-08

**Authors:** Lingxuan Zhu, Jiahua Pan, Weiming Mou, Longxin Deng, Yinjie Zhu, Yanqing Wang, Gyan Pareek, Elias Hyams, Benedito A. Carneiro, Matthew J. Hadfield, Wafik S. El-Deiry, Tao Yang, Tao Tan, Tong Tong, Na Ta, Yan Zhu, Yisha Gao, Yancheng Lai, Liang Cheng, Rui Chen, Wei Xue

**Affiliations:** 1 Department of Urology, Renji Hospital, Shanghai Jiao Tong University School of Medicine, Shanghai 200127, China; 2 Department of Etiology and Carcinogenesis, National Cancer Center/National Clinical Research Center for Cancer/Cancer Hospital, Chinese Academy of Medical Sciences and Peking Union Medical College, Beijing, China; 3 Changping Laboratory, Beijing, China; 4 Department of Urology, Shanghai General Hospital, Shanghai Jiao Tong University School of Medicine, Shanghai, China; 5 Department of Urology, Shanghai Changhai Hospital, Second Military Medical University, Shanghai 200433, China; 6 Department of Surgery (Urology), Brown University Warren Alpert Medical School, Providence, RI, USA; 7 Minimally Invasive Urology Institute, Providence, RI, USA; 8 The Legorreta Cancer Center at Brown University, Lifespan Cancer Institute, Providence, RI, USA; 9 The Legorreta Cancer Center at Brown University, Laboratory of Translational Oncology and Experimental Cancer Therapeutics, Department of Pathology & Laboratory Medicine, The Warren Alpert Medical School of Brown University, The Joint Program in Cancer Biology, Brown University and Lifespan Health System, Division of Hematology/Oncology, The Warren Alpert Medical School of Brown University, Providence, RI, USA; 10 Department of Medical Oncology, National Cancer Center/National Clinical Research Center for Cancer/Cancer Hospital, Chinese Academy of Medical Sciences and Peking Union Medical College, Beijing, China; 11 Faculty of Applied Sciences, Macao Polytechnic University, Address: R. de Luís Gonzaga Gomes, Macao, China; 12 College of Physics and Information Engineering, Fuzhou University, Fujian 350108, China; 13 Department of Pathology, Shanghai Changhai Hospital, Second Military Medical University, Shanghai 200433, China; 14 Department of Pathology and Laboratory Medicine, Department of Surgery (Urology), Brown University Warren Alpert Medical School, Lifespan Health, and the Legorreta Cancer Center at Brown University, Providence, RI, USA; 15 The First School of Clinical Medicine, Southern Medical University, Guangzhou, China

**Keywords:** prostate cancer, artificial intelligence, pathology, machine learning, whole-slide image

## Abstract

Prostate cancer (PCa) is a common malignancy in males. The pathology review of PCa is crucial for clinical decision-making, but traditional pathology review is labor intensive and subjective to some extent. Digital pathology and whole-slide imaging enable the application of artificial intelligence (AI) in pathology. This review highlights the success of AI in detecting and grading PCa, predicting patient outcomes, and identifying molecular subtypes. We propose that AI-based methods could collaborate with pathologists to reduce workload and assist clinicians in formulating treatment recommendations. We also introduce the general process and challenges in developing AI pathology models for PCa. Importantly, we summarize publicly available datasets and open-source codes to facilitate the utilization of existing data and the comparison of the performance of different models to improve future studies.

## Graphical abstract

**Figure:**
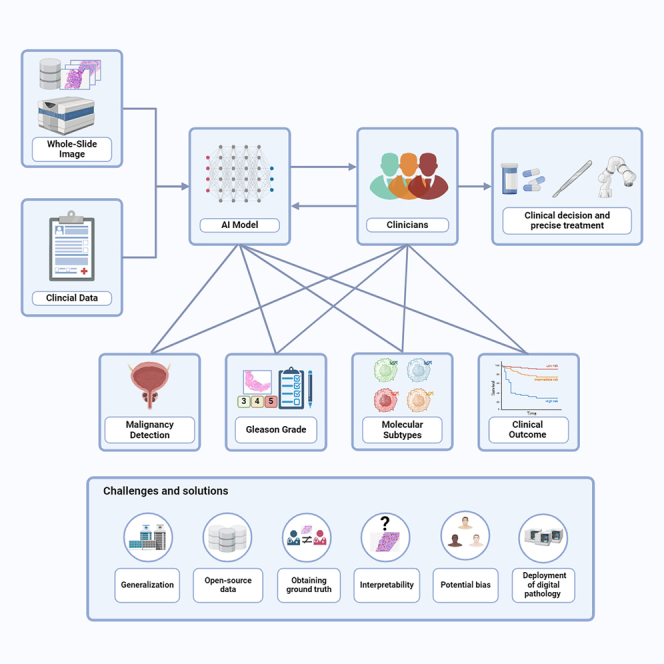

## Teaser

Zhu et al. summarize recent AI advances for prostate cancer management, including automated diagnosis, grading, patient outcome prediction, and molecular subtyping. This review highlights collaborative human-AI systems, public resources, and challenges such as model interpretability, generalization, and bias. AI pathology tools can assist clinicians to enhance efficiency and improve patient care.

## Introduction

Prostate cancer (PCa) is the leading cause of cancer-related deaths in males, with an annual global incidence of approximately 1,414,259 new cases.^1^ Diagnosis typically involves conducting a biopsy after an initial suspicion of PCa based on an elevated prostate-specific antigen (PSA) level. Treatment plans for localized PCa are primarily established based on pathological examination of the biopsy. Pathologists determine whether the patient has PCa and assign a Gleason grade group (GG) by evaluating hematoxylin and eosin (H&E)-stained sections of the tissue.^2^^,^^3^ A multidisciplinary team of urologists, medical oncologists, and radiation oncologists then determine in a collaborative manner based on the biopsy pathology report and radiographic and clinical features whether the patient should pursue active treatment, such as radical prostatectomy (RP) or radiation therapy, or opt for active surveillance (AS). The pathology report following RP is crucial for determining the patient’s postoperative prognosis, predicting the likelihood of biochemical recurrence (BCR), and guiding the selection of adjuvant therapy or other treatment options.^2^^,^^3^

Each patient typically has at least 12 needle biopsies, resulting in over 15 million biopsy samples per year worldwide, highlighting the tremendous workload for pathologists. The shortage of pathologists suggests an even greater workload associated with PCa diagnosis.^4^ In addition, the pathology review of PCa primarily relies on subjective assessments, with reported low inter-observer consistency.^5^^,^^6^^,^^7^ This inconsistency may lead to under-treatment of aggressive cancers and over-treatment of indolent cancers. As a result, researchers are attempting to introduce artificial intelligence (AI) into the pathology review of PCa, aiming to attain expert-level and reproducible outcomes.

AI has demonstrated promising capabilities in medicine, especially in the analysis of medical images.^8^^,^^9^^,^^10^ In PCa pathology, the US Food and Drug Administration (FDA) has approved the Paige Prostate for determining between benign prostate biopsies and suspicious PCa, and six other AI products have received European Conformity (CE) certification (Table 1). This indicates that AI capabilities in PCa pathology analysis have begun to be recognized by regulatory agencies. Nonetheless, the current repertoire of AI products approved for PCa pathology analysis remains limited, and their capabilities are comparatively rudimentary. With the advent of high-throughput automated pathology slide scanners, complete digitization of pathology laboratories is inevitably going to become a reality in the near future.^11^ We believe that AI pathology will have a significant impact on the management of PCa.

**Table 1:** AI tool for pathological analysis of prostate cancer approved by the US FDA or that have received the CE mark

Name of the device	Company	Functions	Country	Related work
Paige Prostate Detect^a^	Paige.AI	detecting tumors in prostate needle biopsies	US	Raciti et al.,^12^ Perincheri et al.,^13^ da Silva et al.,^14^ Campanella et al.,^15^ Raciti et al.^16^
Paige Prostate Grade & Quantify	Paige.AI	Gleason grading and quantification, total tumor percentage, and tumor length measurement	US	Eloy et al.^17^
Aiforia Clinical AI Model for Prostate Cancer	Aiforia	cancer detection and Gleason grading	Finland	Sandeman et al.^18^
DeepDx-Prostate Connect	Deep Bio	ROI detection, Gleason grading, and quantification	South Korea	Jung et al.^19^^,^^20^ and Ryu et al.^19^^,^^20^
Galen Prostate	Ibex Medical Analytics	cancer detection and Gleason grading	Israel	Pantanowitz et al.^21^
HALO Prostate AI	Indica Labs	prostate cancer detection and Gleason grading	US	Tolkach et al.^22^
INIFY Prostate	Inify Laboratories	cancer detection	Sweden	Vazzano et al.^23^

a Approved by the US FDA.

This review focuses on advanced AI research with potential for translation in PCa pathology analysis and emerging research trends, highlighting how AI can collaborate with pathologists and urologists to improve patient care. In addition to common tasks such as automated malignancy detection and automated Gleason grading, we also review cutting-edge developments in predicting patient outcomes, molecular phenotypes, and uncovering possible molecular mechanisms of the disease. We also introduce the general process and challenges in developing AI models in this field. Importantly, we summarize publicly available prostate pathology image datasets and open-source prostate pathology AI model codes, enabling researchers to fully utilize existing data and compare performance against other models, thereby improving the quality of research.

## Development of AI models for prostate cancer management

AI can be broadly divided into two categories: traditional machine learning (ML) and deep learning (DL). The key difference is that the former relies on hand-crafted features (such as color and texture), while the latter utilizes features automatically extracted by convolutional neural networks (CNNs), thus enabling the detection of information that is difficult for humans to obtain. For example, in the development of a DL model for automated Gleason grading, AI learns from annotations provided by pathologists. Different training strategies require different levels of annotation information.^24^ Supervised learning requires pathologists to manually draw every gland on the whole-slide images (WSIs) and provide Gleason pattern (GP) information, which is time consuming. Weakly supervised learning typically uses slide- or patient-level Gleason scores recorded in pathology reports as inputs, without the need for additional annotations. Semi-supervised learning leverages a small set of precisely labeled data alongside larger unlabeled datasets to improve performance. Self-supervised learning generates its own supervisory signal from unlabeled data by defining pretext tasks, then fine-tuning the model on downstream tasks, allowing it to exploit large unlabeled datasets. Generally speaking, these strategies, with the exception of supervised learning, require larger datasets. Weakly supervised learning is currently the most popular strategy.

**Figure 1:**
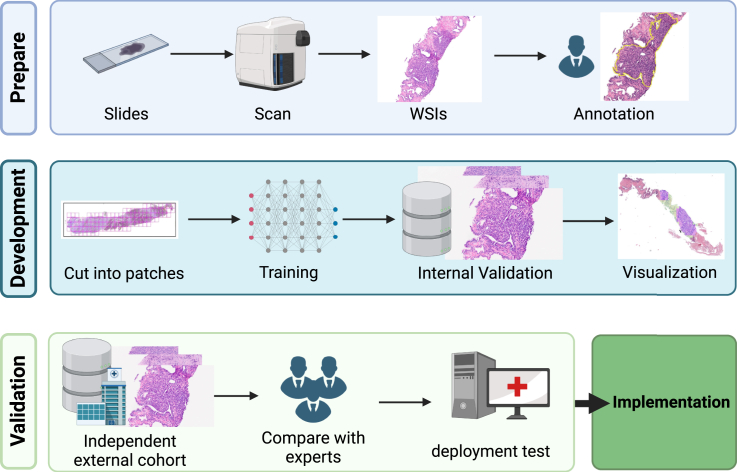
The development process of pathology AI models, using automated Gleason grading as an example

Due to the large size of images, WSIs are preprocessed and split into small patches as model input. After its development, the AI model is validated on independent external validation sets to assess its generalization ability and is compared with human pathologists to determine whether it has reached or exceeded the performance of expert pathologists. Furthermore, prospective deployment testing is conducted to validate its real-world performance and impact on clinical practice. Thus, a prostate AI pathology tool is completed from development to validation and eventually moves toward clinical application (Figure 1).

### AI for the identification of benign and malignant lesions

AI has made remarkable achievements in identifying benign and malignant WSIs (Table 2). One example is Paige Prostate, which has been approved by the FDA for clinical application. This model used multiple instance learning - recurrent neural network (MIL-RNN) technology to train on 12,132 WSIs with the diagnosis of pathology report as labels.^15^ In their study, the model achieved an area under the curve (AUC) of 0.99 on the test set and 0.93 on an independent external validation set of over 12,000 slides.^15^ In a user study, the sensitivity of three board-certified pathologists in diagnosing PCa was significantly improved from 74% to 90% with the assistance of the AI.^12^ The model also performed excellently on an independent external cohort of 600 slides from 100 consecutive patients^14^ and another cohort of 1,876 prostate core biopsy WSIs,^13^ demonstrating its good generalizability.

**Table 2:** Studies on prostate cancer detection and automated Gleason grading models with independent external validation

Author	Purpose	Methods	Type	Dataset size	Performance on external dataset
Vazzano et al.^23^	external validation of the INIFY Prostate model (detection and quantification of tumor area)	CNN	biopsy	external: 30 patients, 30 slides from two centers	detection: specificity 0.97 sensitivity 0.994 quantification of tumor area: correlation coefficient 0.76–0.80
Yang et al.^25^	improving the recognition of small tumor regions (detection of prostate cancer)	intensive-sampling MIL	biopsy	training: PANDA dataset external: HEBEI (100 patients, 844 slides) and DiagSet-B dataset	for all cases, AUC 0.9711 and 0.9822; F1 score: 0.8410 and 0.9709 for hard cases, AUC 0.7348 and 0.9707; F1 score 0.6669 and 0.7957
Xiang et al.^26^	detection and automated GG grading (0–5)	GCN-MIL	biopsy	training: PANDA dataset external: HEBEI (100 patients, 844 slides) and DiagSet-B dataset	detection: AUC 0.985 and 0.986 GG grading^#^: κ, 0.723; κ_quad_ 0.801 (only HEBEI dataset was used for validation in this task)
Oner et al.^27^	detection of prostate cancer	multi-resolution R-CNN-ResNet-18, supervised learning	biopsy	training: PANDA dataset external: 280 cores, 46 patients	AUC: 0.992
Jung et al.^19^	external validation of the DeepDx^20^ model (detection and Gleason grading)	DNN, supervised learning	biopsy	external: 593 slides	detection: accuracy, 0.9831 GG grading^#^: κ 0.713, and κ_quad_ 0.922 GS grading^#^: κ 0.654, and κ_quad_ 0.904 GG1 or normal vs. GG2-5: Accuracy: 0.9376
Singhal et al.^28^	detection and automated GG grading	CNN, semi-supervised learning	biopsy	training: Local (580 slides), PANDA (Radboud) dataset external: PANDA (Karolinska) dataset	detection: AUC: 0.92 GG grading^#^: accuracy: 0.831; κ_quad_ 0.93 GG2 vs. GG3-5: AUC: 0.93
Pohjonen et al.^29^	improve generalization problem for the detection of prostate cancer	neural network trained with spectral decoupling	–	training: 90 patients from Helsinki external: PESO dataset	networks trained with spectral decoupling achieve up to 9.5% point higher accuracy on external datasets (The author did not report the exact value of accuracy.)
PANDA challenge^30^	detection and automated GG grading	various DL algorithms	biopsy	training: PANDA dataset external: two cohorts (714 and 330 slides)	detection: sensitivity 0.986 and 0.0.977; specificity 0.752 and 0.843 GG grading^#^: κ_quad_ 0.862 and 0.868
Silva-Rodríguez et al.^31^	detection and automated Gleason grading	CNN, self-supervised learning	biopsy and RP	training: PANDA dataset external: SICAP, ARVANIT and GERTYCH datasets	detection: sensitivity 0.7568–0.9389; precision 0.9375–0.9720 GP grading^#^: accuracy 0.7226–0.8305; F1 score 0.7319–0.8202; κ_quad_ 0.7930–0.8303 GS grading^#^: κ_quad_ 0.8054–0.8299 GG grading^#^: κ_quad_ 0.8254–0.8854
Mun et al.^32^	detection and automated GG grading	weakly supervised DL	biopsy	training: HUMC 621 cases, 6,071 slides; KUGH 167 cases, 1,529 slides external: Gleason 2019 dataset	inter-institutional∗: detection AUC 0.982 GG grading^#^: accuracy 0.674, κ 0.553, κ_quad_ 0.880 external: detection AUC 0.943 GG grading^#^: accuracy 0.545, κ 0.389, κ_quad_ 0.634
Li et al.^33^	detection of prostate cancer	multi-resolution MIL	biopsy	training: local (830 patients, 20,229 slides) external: SICAP-V1 dataset	AUC 0.994
Silva-Rodríguez et al.^34^	automated GP grading	CNN, supervised learning	biopsy	training: SICAPv2 external: ARVANIT and GERTYCH datasets	GP grading^#^: accuracy 0.5136–0.5861; F1 score 0.4753–0.5702; κ_quad_ 0.5116–0.6410
Nagpal et al.^35^	automated GG grading^†^	CNN, supervised learning	biopsy	training: 360 cases, 524 slides external: 322 slides	agreement rate: 0.801 (all biopsies) and 0.714 (tumor only)
Pantanowitz et al.^21^	detection of prostate cancer and perineural invasion and automated Gleason grading	CNN, supervised learning	biopsy	training: 549 slides external^‡^: 100 consecutive cases, 1,627 slides	detection: AUC 0.991 GS 6 or ASAP vs. GS 7–10: AUC 0.941 GP3–4 or ASAP vs. GP5: AUC 0.971 perineural invasion: AUC 0.957
Tolkach et al.^22^	detection and automated GG grading	CNN, supervised learning	RP	training: TCGA-PRAD dataset external 1^22^: 2 cohorts (592 and 279 patients) external 2^36^: 7473 cores, 423 patients from five centers	external 1: detection: AUC 0.9919 and 0.9918 GG grading^†^: κ 0.51–0.66 external 2: detection: sensitivity 0.971–1.000; specificity 0.875–0.976 grading: κ_quad_ 0.72–0.77
Ström et al. ^37^	detection, measurement of tumor length and automated grading	DNN, supervised learning	biopsy	training: 1,069 patients, 6,953 slides external: Imagebase and Karolinska dataset (330 cores from 73 cases)	detection: AUC 0.986 GG grading: for Imagebase dataset, mean pairwise κ 0.62. for Karolinska dataset^#^, κ 0.70 and 0.76 (after calibrating). cancer length: correlation 0.87
Bulten et al.^38^	detection and automated GG grading	CNN, semi-supervised learning	biopsy	training: 1,243 cases, 5759 slides external: 245 tissue microarray cores	detection: AUC 0.985 GG grading^#^: κ_quad_ 0.715 GG1 or normal vs. GG2-5: AUC 0.875 GG1-2 or normal vs. GG3-5: AUC 0.875
Campanella et al.^15^	detection of prostate cancer	MIL	biopsy	training: 836 patients, 12,132 slides external 1^15^: 6,323 patients, 12,727 slides external 2^13^: 1,876 cores from 118 consecutive patients external 3^14^: 600 cores from 100 consecutive patients	external 1: AUC 0.932 external 2: sensitivity 0.977; PPV 0.979; specificity 0.993; NPV 0.992 external 3: sensitivity 0.99; NPV 1.0; specificity 0.93

Some studies included more than one task; we only collected the tasks that had independent external validation in the table. For specific methodological details, please refer to the original papers. GP, Gleason pattern; GS, Gleason score; GG, Gleason grade group; GG 0, benign; ASAP, atypical small acinar proliferation; NC, non-cancer; AUC, area under the receiver operating characteristic curve; PPV, positive predictive value; NPV, negative predictive value; μIoU, mean intersection over union; κ_quad_, quadratic-weighted kappa; MIL, multiple instance learning, belongs to weak supervised learning; MLP, multi-layer perceptron; GCN, graph convolution network; CNN, convolutional neural network; DNN, deep neural network; HUMC, dataset from Hanyang University Medical Center, Korea; KUGH, dataset from Korea University Guro Hospital; HEBEI, dataset from The Fourth Hospital of Hebei Medical University, China; HEBEI dataset contains 844 WSIs from 100 patients, which are not available to the public. ^#^When reporting performance, the benign category is included. For example, for GG grading, benign and GG1-5 are included for a total of six categories. ∗WSIs from HUMC were used for the discovery, and WSIs from the KUGH were used for the validation. †GG4 and GG5 were combined into one class in this study. ‡Thirty-two cases from this external center were used to calibrate the model prior to external validation.

In contrast to previous studies that used retrospectively collected samples, Ström et al. developed a model based on a prospective, population-based diagnostic study cohort (STHLM3 study) and achieved an AUC of 0.986 in the validation set, even when facing various difficult cases from the real world.^37^ Unlike using AI to directly provide a diagnosis, Dov et al. designed a hybrid human-machine approach in which the AI identified the top 20 regions of interests (ROIs) with the highest malignancy probability for each biopsy. Pathologists were then able to make negative biopsy diagnoses by examining only these ROIs while reserving potentially malignant biopsies for further examination. This approach yielded an outstanding sensitivity of 99.2% and filtered out approximately 70% of all benign WSIs.^39^

Prostate biopsy can sometimes miss cancerous areas, leading to missed diagnosis. Liu et al. hypothesized that there may be tumor-induced morphological changes in benign cores sampled in the vicinity of cancerous regions. They employed CNNs to uncover these subtle morphological variations and successfully detected 70% of GG 5 PCa cases and approximately 30% of low-grade PCa cases by analyzing only the benign biopsy WSIs and incorporating relevant patient clinical information. This can help identify high-risk PCa cases missed by regular biopsies without increasing overdiagnosis risk for low-grade lesions.^40^

### AI for the grading of prostate cancer

Gleason grading is a powerful prognostic factor for PCa and is crucial for treatment decision-making. Automated Gleason grading with AI is practical but challenging. Numerous studies have shown that AI algorithms surpass non-uropathologists and achieve results that are comparable to those of expert-level uropathologists^19^^,^^37^^,^^41^^,^^42^^,^^43^ (Table 2).

Numerous AI challenges have promoted development in this field, with the most influential being the PANDA Challenge.^30^ Algorithms developed using 10,616 prostate biopsy samples from multiple centers achieved expert-level performance in two independent cross-continental validation sets, reaching concordance of 0.862 and 0.868 with expert uropathologists, demonstrating a robust performance of the AI models that is comparable to that of pathologists.^30^ Some researchers have proposed fine-grained Gleason grading concepts, such as using the prediction scores of DL system to subdivide the GP into GP 3.5 and GP 4.5, which achieved more accurate risk stratification.^43^

On the other hand, as current technology cannot perfectly assign each patient to a specific group, many studies opt to divide patients into low-grade or high-grade (GG1 vs. GG2-5 or GP 3 vs. GP ≥ 4) based on clinical treatment implications,^2^^,^^21^^,^^35^^,^^38^^,^^42^^,^^44^^,^^45^^,^^46^^,^^47^^,^^48^ representing whether patients are suitable for AS or RP. For example, Pantanowitz et al.'s model achieved an AUC of 0.941 in distinguishing between high- and low-grade PCa in an external validation set.^21^ In another study, Bulten et al.'s model achieved AUCs of 0.878 and 0.869 in distinguishing between benign and GG 1 vs. GG ≥ 2 in two external validation sets.^38^

As the field progresses, weak supervision technology has emerged as a promising solution to free pathologists from time-consuming pixel-level annotation and fully leveraging the large-scale diagnostic labels available in pathological reports.^15^^,^^31^^,^^32^^,^^33^^,^^49^^,^^50^^,^^51^^,^^52^ Additionally, various new techniques have been proposed to improve AI model performance, such as knowledge distillation, deep quantum ordinal regression, and pyramid semantic parsing network.^53^^,^^54^^,^^55^

## AI applications beyond diagnosis and grading

AI can effectively tackle various tasks beyond diagnosis and grading. For example, automatic measurement of cancer length and volume,^20^^,^^23^^,^^37^^,^^56^ quantification of GP percentage,^43^^,^^57^^,^^58^ recognition and quantification of perineural invasion,^21^^,^^59^ quantification of immunohistochemistry (IHC) staining,^60^^,^^61^ and detection and quantification of cribriform pattern.^34^^,^^62^^,^^63^ AI models have also been developed to assess tumor purity of PCa using frozen H&E-stained slides.^64^ AI has significantly improved consistency and reproducibility of these tasks. AI can also streamline pathology laboratory workflows by identifying ambiguous cases that require additional IHC-stained examination and automatically requesting this before pathologist review, thus reducing turnaround times.^65^ AI can also perform fully automatic quality control of WSI scans and identify scans that may require re-scanning or re-staining to improve quality.^66^ In a cutting-edge field, Rana et al. attempted to add computationally generated H&E staining to unstained prostate biopsy images and found that the algorithm-generated images could accurately replicate prostate tumor characteristics and be used for pathological diagnosis, thus supporting early detection of abnormalities in non-stained tissue biopsies.^67^

## Empowering pathologists: AI as a collaborative tool

**Figure 2:**
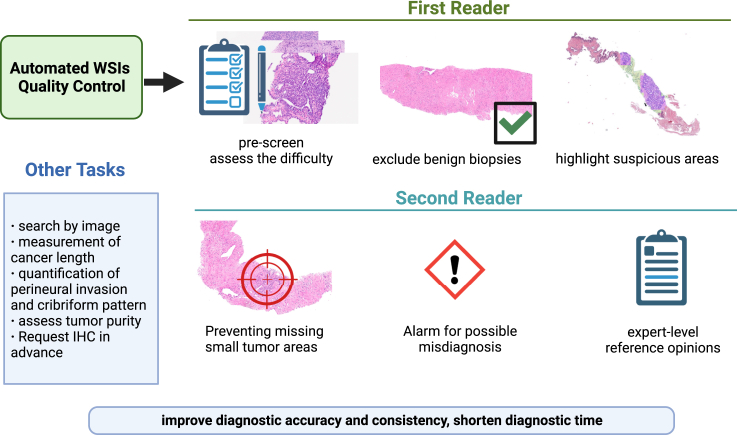
Interaction between pathology AI models and pathologists

Due to legal, ethical, and other considerations, it is unlikely that AI will replace human pathologists in the near future. However, AI can assist in standardization and quality control in pathological analysis^68^^,^^69^^,^^70^ (Figure 2). Multiple studies have shown that the performance of AI in conjunction with human pathologists is superior to either alone, and it can improve diagnostic accuracy and consistency, shorten diagnostic time, and reduce over-grading and over-treatment.^12^^,^^57^^,^^71^^,^^72^ Since a considerable proportion of prostate biopsies are benign,^73^ AI as a first reader can help pathologists pre-screen and exclude benign biopsies, allowing them to focus on those with higher risk of malignancy. AI can also assist pathologists in quickly locating suspicious areas, thus improving diagnostic speed. Eloy et al. found that, with the assistance of AI, pathologists can reduce need for IHC and second opinions while maintaining accuracy.^17^ AI can also assess the difficulty of a case and prioritize difficult samples to be assigned to expert-level pathologists.^74^

As a second reader, AI serves as an unbiased reviewing tool, marking samples that differ in results between pathologists and AI,^13^ and preventing pathologists from missing small tumor areas.^15^^,^^16^^,^^28^ In one study, AI assistance increased the sensitivity for detecting prostate cancer smaller than 0.6 mm from 46% to 83%.^12^ Pantanowitz et al. developed a system that alerts pathologists to slides where AI detects potential cancer that was missed by the pathologist or indicates slides that were diagnosed as Gleason score (GS) 6 by the pathologist but had a higher GS determined by AI.^21^ This system allows pathologists to view WSIs with AI-flagged regions for re-review, and it successfully identified a missed prostate cancer case during real-world deployment.^21^ Furthermore, AI systems with expert-level performance can provide general pathologists with expert-level reference opinions to assist in diagnosing challenging cases.^19^^,^^72^ Therefore, the second review provided by AI can improve diagnostic consistency, help achieve standardized grading, and bring expert-level grading to areas with limited medical resources.^37^^,^^75^

AI can also enhance the utility of histopathology image datasets within a medical institution by providing a “search-by-image” function. Pathologists can match images from the dataset to the current patient and relevant pathological reports for decision support. This is especially helpful for rare or unusual cases.^76^^,^^77^^,^^78^

### AI for prediction of prognosis

One of the primary purposes of Gleason grading is to predict patient prognosis. Some studies have found that AI’s GSs are more effective in stratifying patient risk.^22^^,^^42^^,^^43^ In addition, Wulczyn et al. developed a model based on WSIs from RP specimens to quantify the percentages of GP4 and GP5. They then used a Cox proportional hazards regression model based on the obtained GP percentages to generate an AI risk score for predicting prostate cancer-specific mortality. The model achieved a C-index of 0.87, outperforming the predictive ability of the GG system (C-index of 0.75) and effectively stratified patient outcomes.^79^ These results demonstrated that AI could surpass the accuracy of commonly used clinical models for risk prediction and stratification.

Furthermore, the goal of AI should not be limited to replicate pathologists' assessments. Pathology slides contain a large amount of biological information not covered by the Gleason grading system. Instead of having AI learn the inherently biased Gleason grading system, it may be better to train AI directly on WSIs using long-term follow-up data to guide risk stratification.^80^^,^^81^ For example, Esteva et al.^82^ developed a model based on WSIs from PCa patients, achieving outstanding performance in predicting prostate cancer-specific mortality and 5-year distant metastasis events with AUCs of 0.77 and 0.83, respectively.

Biochemical recurrence (BCR) is a clinical endpoint that patients with PCa may encounter in a relatively short time. AI has achieved promising results in directly predicting BCR from WSIs, outperforming traditional clinical indicators such as GS in several studies.^83^^,^^84^^,^^85^^,^^86^^,^^87^^,^^88^^,^^89^^,^^90^ For instance, Yamamoto et al. applied an unsupervised deep neural network (DNN) model to extract features from unannotated post-RP H&E slides to predict 1-year BCR, achieving an AUC of 0.845 in an external validation set, which was better than using only GS (AUC = 0.721).^87^ Interestingly, combining AI with GS yielded the best performance (AUC = 0.884).^87^

AS rather than RP is becoming the preferred management approach for low-risk PCa.^91^ Tsuneki et al. developed a DL model to determine whether patients should choose AS or RP based on the proportions of GP 4 and 5, achieving an AUC of 0.84 for identifying indolent tumors suitable for AS.^92^ However, accurately identifying AS patients at higher risk of disease progression remains challenging. Chandramouli et al. used WSIs from needle-core biopsy to predict progression in AS patients, achieving an AUC of 0.75, outperforming pro-PSA and Gleason.^93^ AI can also predict other PCa prognosis indicators like lymph node metastasis (AUC = 0.68),^94^ the development of castration-resistant prostate cancer (CRPC) after combined androgen blockade (CAB) therapy (sensitivity and specificity both 87.5%),^95^ and adverse pathology outcomes (AUC = 0.72).^96^ However, relying solely on morphological analysis may be insufficient for long-term outcome predictions, as randomness of mutations and the potential for escaped cells to acquire additional mutations can affect recurrence.^68^^,^^86^

### AI for prediction of molecular subtypes

In addition to pathological features, genetic sequencing has identified molecular markers associated with PCa progression. However, the cost and complexity have limited its clinical application. Genetic changes in tumor cells can lead to changes in their function, which can in turn affect the morphology of tumor cells.^97^ The connection between the changes in tumor molecular composition and morphology can be found in almost every cancer and nearly every class of molecules,^98^ providing a foundation for using AI to predict molecular characteristics from WSIs. Compared to genomic biomarkers, AI-based digital pathology systems are easier to obtain the input, more cost-effective, and more scalable for clinical application. For example, TMPRSS2-ERG gene fusion is a commonly observed genetic alteration in PCa, which is associated with aggressive PCa^99^ and correlates with certain morphological characteristics of PCa.^100^ Dadhania et al. established a CNN-based model to predict the presence of TMPRSS2-ERG gene fusion with WSIs of PCa patients, with an AUC of 0.82–0.84.^101^ Similarly, Erak et al. used a self-supervised DL model to predict ERG gene fusion and PTEN loss status with average AUCs of 0.83 and 0.76 over multiple independent external validation sets.^102^ The model can even predict the spatial patterns of subclonal PTEN deletions within tumor nodules. The SPOP mutation is a specific subtype of PCa. Schaumberg et al. used a ResNet model to determine the SPOP mutation status in PCa patients, achieving an AUC of 0.7589 in an external validation cohort.^103^ Furthermore, a pan-cancer DNN model can predict TP53 and FOXA1 mutation status in PCa patients based on WSI, with AUCs of 0.685 and 0.762, respectively.^97^

Furthermore, AI can predict gene expression from pathology images. Weitz et al. proposed a multioutput CNN model based on gene co-expression patterns to predict gene expression in PCa from WSIs. This model identified associations between morphology and gene expression for 5,419 genes.^104^ To assess the clinical utility of the model, they calculated the CNN model’s Prolaris Cell Cycle Progression (CCP) score, which is a commercial prognostic test based on gene expression values to assess the risk and progression of PCa. The CNN-derived CCP score achieved an AUC of 0.73 when discerning whether the RNA sequencing (RNA-seq)-based CCP score was above or below the median. In summary, the utilization of AI for predicting the molecular subtype of PCa can be particularly beneficial in low-resource settings, where molecular diagnostics are not readily available. However, the lack of large-scale PCa cohorts with both high-quality WSIs and corresponding genomic sequencing data poses a challenge for further validating the potential of AI for this task.

AI can also be applied to uncover connections between morphological changes and potential molecular alterations. Huang et al. used an AI model’s morphological scoring system to identify ROIs associated with early recurrence of PCa.^89^ Subsequently, multiplexed IHC was used to compare the expression of biomarkers within the high- and low-scoring ROIs identified by the AI model. They discovered that the high expression of TMEM173 within high-scoring ROIs may be a potential biomarker for driving early recurrence in PCa patients.^89^ We believe that by further combining spatial omics techniques, we could uncover more molecular mechanisms behind the ROIs identified through automated AI feature extraction. Additionally, spatial omics annotates each region of pathology images with corresponding genetic information, which potentially refines AI’s accuracy in predicting gene mutations and expressions. This advancement could also empower AI models to predict the spatial distribution of diverse molecular subtypes within tumors, thus providing deeper insights into intratumoral heterogeneity (ITH). AI can also help us understand ITH by visualizing the distribution of different GPs within the tumor and identifying patterns of tumor-infiltrating lymphocyte (TIL) distribution.^105^ In conclusion, AI has the ability to go beyond simple morphological analysis and can extract more information from pathology slides than humans.

## Challenges of application of AI in clinic

### Difficulty in generalization

Owing to the heterogeneity among datasets and the risk of overfitting, AI models may perform less effectively when applied to new datasets, limiting their widespread use.^30^^,^^50^ Heterogeneity can stem from various factors, such as staining variations, artifacts, and imaging differences between scanners.^29^^,^^75^^,^^106^^,^^107^ To overcome this, an ideal approach is to have a sufficiently large and diverse training set, such as continuously collecting all cases over a certain period of time from multiple institutions, in order to cover all possible variations in the real world to represent the entire target population. From the perspective of fully utilizing the existing data, data augmentation techniques such as rotation, flipping, and color enhancement can be applied to enhance the original training set.^48^^,^^51^ Alternatively, introducing histological artifacts into the training data^107^ or fine-tuning model parameters using a subset of samples from the new dataset can help improve the generalization ability of the model.^61^ Additionally, tools such as color ^108^^,^^109^ or style normalization^110^ can make new data more similar to the training set. For example, after using style normalization, the AUC of the AI model on an independent external validation set increased from an average of 0.875 to 0.975, which is close to the results of the training set cross-validation (AUC = 0.98),^110^ indicating that the generalization gap of the model can be compensated for by appropriate preprocessing. In summary, these methods can strengthen the generalization ability of AI models in the real world.

### Lack of high-quality open-source data

Unlike many other medical imaging techniques, routine pathology workflows are rarely fully digital, requiring additional scanning to obtain WSIs. Moreover, publicly available datasets for PCa WSIs are scarce and stored sporadically, hindering AI progress in this area. AI models applied to PCa pathology analysis should be evaluated on independent test sets from different institutions; otherwise, it is difficult to assess whether AI can perform well in complex external clinical scenarios.^111^ Despite an increasing number of relevant articles in recent years, the deployment of AI tools in clinical practice remains limited (Table 1). Many studies focused solely on algorithmic improvement using data from a single institution, lacking external validation or comparison with human pathologists. Additionally, variations in datasets and metrics used to evaluate model performance hinder direct comparisons among different studies. Therefore, we summarized the publicly available WSIs datasets of PCa (Table 3) and the source code of several AI models (Table S1), facilitating model validation with external datasets and enabling comparisons with established models.

**Table 3:** Publicly available prostate pathology image datasets

Dataset	Data type	Sample type	Dataset size	Support data
PANDA Challenge^30^^,^^112^	WSI	biopsy	2,113 patients, 10,616 slides	annotations of stroma, benign, and GPs 3, 4, and 5 for data from Radboud; benign and cancerous tissue for data from Karolinska
TCGA-PRAD^113^	WSI	RP	403 patients, 449 slides	clinical, various sequencing data, pathological report
SICAP-MIL^49^	WSI	biopsy	271 patients, 350 slides	annotations of GPs WSI-level GS including both primary and secondary GPs
Ibex^21^	WSI	biopsy	210 patients, 2,501 slides	associated IHC slides
PESO^114^^,^^115^	WSI	RP	102 patients	annotations of benign and cancerous tissue
DiagSet^116^	WSI and Patches	biopsy	5,151 slides	set A: patch-level annotations for 2.6 million patches extracted from 430 WSIs set B: slide-level annotations of cancer or non-cancer set C: slide-level annotations of cancer, non-cancer, or need IHC from nine pathologists
NADT-Prostate^117^	WSI	biopsy and RP	39 patients, 1,404 slides	clinical, exome, genome, RNA-seq, slides stained with antibodies against p53, PTEN, AR, PSA, GR, Ki67, SYP, and PIN4-cocktail (p63 + CK5 + K18 + AMACR)
Gallo^118^	WSI	biopsy	167 patients, 787 slides	annotations of tumor regions
PAIP^119^	WSI	RP	600 slides	not reported
AGGC22^120^	WSI	biopsy and RP	241 slides	pixel-level annotations of GPs 38 slides scanned by multiple scanners
PathPresenter^121^	WSI and TMA	biopsy and RP	208 slides	slide-level diagnosis
Prostate Fused-MRI-Pathology^122^	WSI	RP	16 patients, 114 slides	annotations of cancer presence, MR
Tolkach^36^	WSI	biopsy	100 biopsies from two centers	GG grading from 10 or 11 pathologists
Oner^27^	WSI	biopsy and RP	99 slides	GS
PAIP 2021 Challenge^123^	WSI	RP	80 slides	annotations of perineural invasion
STHLM3^37^^,^^124^	WSI	biopsy	60 slides are available	Gleason group, predictions of GPs generated by the AI
CMB-PCA^125^∗	WSI	biopsy	eight patients, 10 slides	clinical, CT, MR, NM, genomic, phenotypic
Zhong^126^	TMA and WSI	RP	71 cores and two WSIs	clinical, PTEN DISH
Tolkach^22^	patches	RP	592 patients from Charite University Hospital and 279 patients from UKB	patch-level annotations of benign or cancerous tissue
Schömig-Markiefka^107^^,^^127^^,^^128^	patches	not reported	15, 18, and 51 patients from three centers, respectively	Each dataset contains 50,000 patches with tumor tissue, 50,000 patches with nonneoplastic glandular prostate tissue, and 20,000 patches with nonglandular tissue.
Prostate-MRI^129^	patches	RP	26 patients	MR
Arvaniti^42^^,^^130^	TMA	not reported	886 cores from five TMAs	clinical, annotations of GPs from two pathologists
Gleason 2019 Challenge^131^	TMA	RP	231patients, 331 cores	annotations of GPs from six pathologists
RINGS^132^^,^^133^	Images	biopsy	1,500 images extracted from WSIs of 150 patients	annotations of the contours of the glands
Gertych^134^	images	RP	210 images extracted from WSIs of 20 patients	annotations of stroma, benign epithelium, GP 3 or 4
Imagebase^135^	images	biopsy and RP	120 cases	GS reviewed by members of an international panel of 24 experts in each of the main fields of urological pathology
Kweldam^136^	images	not reported	60 cases (in supplemental information)	23 genitourinary pathologists’ annotations of predominant GP per case (3, 4, or 5), and to indicate the predominant GP 4 growth pattern, if present

PRAD, prostate adenocarcinoma; RP, radical prostatectomy; WSI, whole-slide imaging; TMA, tissue microarray; NM, nuclear medicine; UKB, The University Hospital Bonn. ∗The dataset is ongoing. Note: different datasets have different terms of use. You should comply with the respective terms of use before conducting research.

### Difficulty in obtaining ground truth

Owing to inter-observer heterogeneity in the annotation of prostate WSI data among experts, it is difficult to obtain a perfect set of labels in practice. To ensure the label quality, a committee of experts is often relied upon.^137^ Training and evaluating an AI model based solely on one pathologist’s annotations may result in inaccurate ground truth or bias toward that pathologist’s grading habits, leading to poor performance in external evaluations and limiting its ability to surpass the performance of that expert.^30^^,^^45^ Therefore, it is beneficial to train AI models on datasets with annotations from multiple experts, such as Imagebase^135^ and PANDA,^30^ enabling the model to learn more accurate ground truth.^45^ Furthermore, evaluating the performance of the model by comparing it with annotations from multiple experts enables a more realistic assessment of its capabilities.^45^ When faced with disagreements in expert annotations, the majority voting method is typically used to determine final labels,^19^ and the STAPLE algorithm can be used to merge annotations from multiple experts to construct the final ground-truth label^48^^,^^55^ Some studies also employed automatic label-cleaning methods to refine annotations iteratively.^30^ Additionally, Arvaniti et al. found that, due to the limited accuracy of manual annotations, pathologists sometimes include stromal tissue in the training area when annotating GP 3, causing AI to misidentify stromal regions as GP 3 during predictions.^42^ To improve annotation accuracy, it would be beneficial to pre-segment the glandular regions and then allow pathologists to specify the GPs.^115^

### Poor interpretability of AI

One major drawback of AI pathology is its lack of interpretability. Many researchers attempted to enhance the interpretability of their models.^138^ One common method is to use gradient-weighted class activation mapping (Grad-CAM) to visualize the ROI that the AI model focuses on.^42^ Another method involves pathologists reviewing AI model feature clustering results. In a study on predicting BCR, pathologists discovered that the model identified features of non-cancerous stroma as prognostic factors after reading representative features, which are not typically evaluated in prostate pathology analysis.^87^ Pinckaers et al. used automatic concept explanations (ACEs) to explain which image features in their model were used to make decisions for predicting BCR.^86^ Therefore, enhancing the interpretability of AI models may discover new histopathological features and increase our understanding of this disease.

In addition, introducing the common errors and advantages of AI to pathologists can help them better handle the predictions given by AI and alleviate potential over-reliance.^16^^,^^47^^,^^57^ Toledo-Cortés et al. quantified the uncertainty of AI predictions in their model, avoiding the limitations of previous models that only provided final prediction results. This can serve as a quality control tool for AI-based diagnoses, allowing pathologists to decide whether to trust the model’s prediction.^54^^,^^80^

### Difficulty in tackling rare conditions, tumor heterogeneity, and ethnic variability

Although most PCa are acinar adenocarcinomas, there are many rare cases and confounding factors in clinical practice, such as inflammation, atrophy, atypical small acinar proliferation, atypical intraductal proliferation, and some diagnostically challenging histological subtypes (such as ductal and intraductal carcinoma of the prostate, prostatic adenocarcinoma with neuroendocrine differentiation), and treatment-related changes. During AI training, most studies excluded these rare conditions, or had limited cases, posing challenges when encountering such cases in real-world scenarios.^13^^,^^16^^,^^30^^,^^33^^,^^39^^,^^68^^,^^72^^,^^139^ Some researchers have proposed using generative adversarial network (GAN) technology to synthesize high-fidelity pathological images to compensate for the small sample size of certain types in the training dataset.^64^^,^^140^ Falahkheirkhah et al. employed this technology to synthesize PCa pathology images, and they mixed synthetic images with real images to train a model that classifies prostate tissue into epithelial and non-epithelial classes.^141^ The model outperformed the model trained using only real data on independent test data. In addition, pathologists were unable to distinguish between real and synthetic images in their study. Additionally, most studies rely on prostate biopsies to train and validate AI models (Table 2), but other tissue samples (e.g., transurethral resection or RP specimens) differ from biopsy tissue in many characteristics, raising questions about the accuracy of models trained on biopsy samples for other types of samples.

Tumor heterogeneity is another issue that needs attention. In clinical practice, more than one slide will be generated, whether it is a prostatectomy or biopsy specimen. However, most studies simply selected a representative slide per patient for training or validation. PCa is a highly heterogeneous tumor with multiple lesions presenting with different GPs. This especially poses challenges for predicting molecular subtyping and prognosis. Future research should incorporate data from all available slides of a patient. It would be of value to compare results generated from analyzing all slides versus single slides with the highest histologic grade.

Another key point is ensuring that the model is universally applicable to all patients, including specific subsets based on age, race, nationality, or other factors.^142^ For example, there are biological differences in PCa among races. Previous study found that the Asian population has a significantly higher frequency of FOXA1 mutations than European and American populations.^143^ Currently, large international studies are limited to populations in Western countries.^30^ Therefore, future investigation is warranted to explore the cross-ethnic applicability of AI pathology models to ensure that the models do not introduce discrimination or bias into clinical practice.^69^^,^^80^

### Scaling up the deployment of digital pathology

Clinical deployment of AI requires digital pathology labs; however, currently only a small portion of laboratories are fully digitized, even in high-resourced countries. To realize the benefits of computational pathology worldwide, we need to ramp up digital slide scanner availability and analysis capabilities cost-effectively. Pathology laboratories at regional medical centers could be prioritized for digitization, serving patients from their own institutions while also providing expert consultation services for other local institutions. Pathology laboratories could also choose to only scan a subset of challenging cases for AI solutions and teleconsultation before full digitization. This would allow patients with more intricate medical conditions to benefit from AI. As technology advances and economies of scale are achieved, the costs of deployment will decrease. Meanwhile, interim solutions such as computational-capable microscopes can fill the gap until whole-slide imaging catches up globally. For example, Chen et al. proposed an augmented-reality microscope (ARM) that overlays AI-based information in real time onto the current view of the sample, seamlessly integrating AI into routine workflows with relatively low-cost retrofitting.^144^

### Limitations of AI and the impact of AI on the behavior of pathologists

Despite AI’s significant progress, potential flaws or biases may still lead to missed or incorrect diagnoses. Additionally, the misdiagnoses made by AI are not entirely without merits. In Tolkach’s study, pathologists recognized false-positive alerts from AI as useful warnings, as these highlighted areas required additional attention and immunostaining for further evaluation.^36^ However, there is a risk that pathologists may overly rely on AI predictions without critically evaluating its predictions. Meyer’s small-scale study showed that pathologists were willing to trust AI regardless of its accuracy.^138^ This raises the question of whether AI would lead to overdependence and compromise pathologists' diagnostic skills. Therefore, similar to post-marketing surveillance for new drugs, continuous monitoring of pathology labs deploying AI tools is essential. This involves understanding how pathologists handle the results generated by AI and observing the impact of AI on clinical practice.

## Prospectives

Recent advances in DL have brought AI to a level comparable to that of pathologists. AI has shown great potential in distinguishing between benign and malignant tumors, automated Gleason grading, and the prediction of clinical prognosis and molecular subtypes. These tools can stratify patient risks and assist urologists in making clinical decisions. However, deploying AI systems in practice requires that AI systems be accurate and trustworthy and not contain biases or flaws that could lead to incorrect diagnoses or inappropriate treatment recommendations. Thus, efforts should focus on improving the generalizability of pathology AI and bridging the gap between regulatory testing and real-world clinical practice datasets. This entails prioritizing robust AI systems that are well-designed, rigorously tested, and continuously monitored for accuracy. Collaboration between AI experts, pathologists, and regulatory bodies is essential, along with ongoing training to effectively integrate pathology AI into practice.

This review provides an overview of the current developments and applications of AI in PCa management. One promising direction for future research is to predict molecular subtype based on pathology images or combining it with other omics techniques for precise diagnosis and treatment recommendations. In addition, generative AI tools such as ChatGPT also hold promise for future research. Drawing from its success in other fields, tools such as ChatGPT can be fine-tuned for specific tasks in PCa management, such as simplifying pathology reports for patient understanding.^10^^,^^145^ Integrating patient histories with WSIs for diagnosis and treatment recommendations is also feasible with updated multimodal analysis capabilities.^146^^,^^147^ However, careful evaluation is necessary to avoid hype and exaggeration.

## Data and code availability

Not applicable.

## Supplemental information

Table S1. Open-source AI models for pathological analysis of prostate
